# The Engineering Side of Hospital Work

**Published:** 1905-12-09

**Authors:** 


### THE ENGINEERING SIDE OF HOSPITAL WORK.

### XVI.—APPARATUS FOR GENERATING ELECTRICITY.

Dynamo Electric Machines.

The dynamo electric machine, or the dynamo as it is usually called, is based upon the principle mentioned in a former article, that when a conductor passes across a magnetic field an electric pressure is created in the conductor, of such a nature that the current which will pass, in obedience to the pressure created, resists the motion. That is to say, the current that is generated in the conductor is such as will create a magnetic field that will cause attraction between the conductor and the primary field, that across which the conductor is passing. It may be noted that this fact is of great importance in connection with the passage of electric currents through the tissues of the human body. Though, when an electric pressure is applied to two points or surfaces on the body, currents will pass between those points or surfaces by every path open to them, the major portion of the currents will pass by the blood in the large blood-vessels. If we take, for simplicity, the case of a pressure applied between one hand and one shoulder, there are two large blood-vessels lying between, one carrying arterial and the other venous blood, the course of the blood being opposite in the two. Each current passing in each blood-vessel will create its own magnetic field around it, the field taking the form of concentric circles surrounding the blood-vessel. The motion of the blood in each vessel will be across the magnetic field created by the current passing in the other vessel, and there will be created in the blood in each vessel an electric pressure tending to oppose the movement of the blood. Further, when, as with alternate currents, the direction of the field is changed fifty or more times in each second, somewhat complicated actions will be set up. The writer will leave the matter there for the present, promising, with the Editor's permission, to thrash it out thoroughly later on. He will now pass to the construction of dynamo machines. These are masses of iron, upon parts of which coils of copper wire are wound, the whole being arranged in such a manner that a very powerful magnetic field is created within a cylindrical space, in which the conductors which are engaged in generating the electric pressures are moved. Another point had better be mentioned here.

Magnetic Resistance, or Reluctance.

The latter term is sometimes used to distinguish it from electrical resistance. As with electricity, all bodies conduct the lines of force which denote the presence and measure the strength of the magnetic field, and all bodies oppose their passage. Of all the substances, iron conducts magnetism with greater facility than any other. A few of the metals—nickel, cobalt, and some of the rarer metals—also conduct magnetism with comparative ease, but the bulk of the other substances offer about the same resistance—namely, 1,460 times that of iron. And this leads to the lines of force created by any magnetic field concentrating very largely in any iron masses that lie in their path. There is another substance which also offers a comparatively low resistance to magnetism that is of consequence in the passage of electricity through the body, and that is oxygen gas. This will also be dealt with later on. Generally, the modern dynamo machine, whether for generating alternate currents or continuous, and whether for one, two, or three phases, consists of a hollow cylinder of iron, on the inner surface of which are carried electromagnets, projecting inwards and carrying crescent-shaped polepieces, the polepieces forming together a nearly complete cylinder. Within this cylinder a cylinder of iron, nearly solid, is carried, having coils of copper upon its surface. This is the armature, and the office of the iron cylinder is to direct the lines of magnetic force, created by the electromagnets on the inner surface of the outer cylinder, into itself, so that there shall be an intense field in the space between the armature and polepieces, and that the copper conductors carried on its surface shall cut the lines of force as the armature revolves, and so generate electric pressures. In the modern dynamo both the iron cylinder forming the core of the armature, and the iron which forms the cores of the electromagnets creating the magnetic field, the field magnets, as they are called, are built up of thin plates of iron, the object of this being to prevent the generation of currents in the iron itself. The drum forming the armature core also has slots cut in its surface, and in these the copper coils are laid, the copper and iron being well insulated from each other. The coils in which the currents pass for the field magnets are carried on what are termed bobbins, which are slipped over the iron cores, and are kept in place by the form of the polepieces. The armature is carried upon a shaft, which runs in bearings supported by pedestals fixed upon the baseplate of the machine. In the latest forms of alternate current machines the construction is slightly altered; the coils on the inner surface of the outer cylinder are those forming the armature, and the field magnets are carried on the ends of arms of a wheel, the effect being the same—the creation of a powerful magnetic field between the magnet poles and the armature coils. The revolution of the field magnets in front of the armature coil is the equivalent of the revolution of the armature in front of the field magnets.

*(To be continued.)*

